# Review of the Two-Step H_2_O/CO_2_-Splitting Solar Thermochemical Cycle Based on Zn/ZnO Redox Reactions

**DOI:** 10.3390/ma3114922

**Published:** 2010-11-12

**Authors:** Peter G. Loutzenhiser, Anton Meier, Aldo Steinfeld

**Affiliations:** 1 Department of Mechanical and Process Engineering, ETH Zurich, 8092 Zurich, Switzerland; E-Mail: peterloutzenhiser@ethz.ch (P.L.); 2 Solar Technology Laboratory, Paul Scherrer Institute, 5232 Villigen PSI, Switzerland; E-Mail: anton.meier@psi.ch (A.M.)

**Keywords:** solar, energy, concentrated, thermochemical cycle, hydrogen, syngas, reactor

## Abstract

This article provides a comprehensive overview of the work to date on the two‑step solar H_2_O and/or CO_2_ splitting thermochemical cycles with Zn/ZnO redox reactions to produce H_2_ and/or CO, *i.e.,* synthesis gas—the precursor to renewable liquid hydrocarbon fuels. The two-step cycle encompasses: (1) The endothermic dissociation of ZnO to Zn and O_2_ using concentrated solar energy as the source for high-temperature process heat; and (2) the non-solar exothermic oxidation of Zn with H_2_O/CO_2_ to generate H_2_/CO, respectively; the resulting ZnO is then recycled to the first step. An outline of the underlying science and the technological advances in solar reactor engineering is provided along with life cycle and economic analyses.

## Nomenclature

*A* = area

*C* = solar flux concentration ratio

*c*_p_ = heat capacity

*E*_a_ *=* apparent activation energy

*G* *=* Gibbs free energy

*H =* enthalpy

*I* = direct-normal solar irradiance

*k =* rate constant

*k*_eff_ = effective thermal conductivity

*k*_0_ = pre-exponential factor

*M* = molecular weight

*m* = mass

*n* = molar amount

$\dot{n}$ = molar flow rate

*Q* = heat transfer rate

$q^{\prime \prime \prime }$ = volumetric heat sink

*R =* universal gas constant

*$r^{\prime \prime \prime }$ =* rate of reaction

*S* = surface area

*t* = time

*T* = absolute temperature

*T_L_* = temperature of surroundings

*T*_R_ = nominal reactor temperature

*W* = rate of work

*y* = mole fraction

*α =* order of reaction

*η* = efficiency

*ρ* = density

*σ* = Stefan-Boltzmann constant

## 1. Introduction

Solar thermochemistry uses concentrated solar energy as the source of high-temperature process heat to drive endothermic reactions. It provides an efficient route for storing intermittent solar energy in the form of chemical fuels such as H_2_ and CO. The combination of H_2_ and CO constitutes synthesis gas (syngas), which is the precursor to renewable liquid hydrocarbon fuels required to power the transportation sectors. Using sunlight to produce syngas directly from H_2_O and captured CO_2_ signifies a promising path towards sustainable energy utilization.

**Figure 1:**
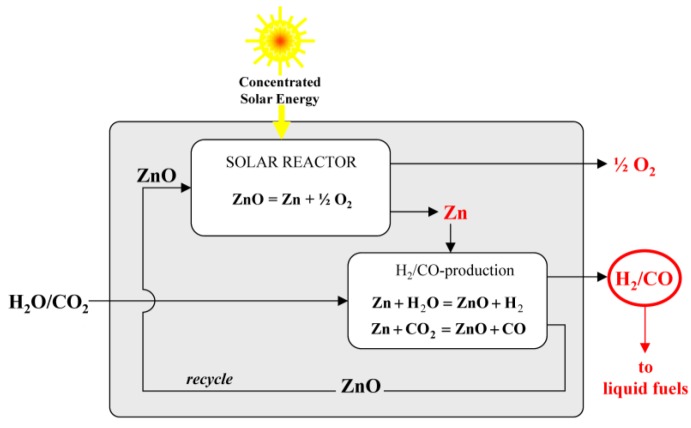
Schematic of the two-step solar thermochemical cycle based on Zn/ZnO redox reactions to produce H_2_ and/or CO from H_2_O and/or CO_2_.

The direct solar thermolysis of H_2_O [1,2] and CO_2_ [3,4,5] require ultra-high temperatures (>2,500 K) at which gaseous products must be separated to avoid recombination or resulting in an explosive mixture, making their realization difficult. The required process temperature can be reduced and the separation problem by-passed via two-step thermochemical cycles based on metal oxide redox reactions [6,7,8,9,10,11,12,13,14]. The Zn/ZnO redox pair emerges as one of the most promising for solar-driven H_2_O and CO_2_ splitting cycles [15,16,17,18], shown schematically in Figure 1.

The first step is the thermal dissociation of ZnO using solar process heat, represented as:

(1) $$ZnO\to Zn+0.5O_{2}$$

The second non-solar step is the exothermal reaction of Zn with H_2_O and/or CO_2_ to generate H_2_ and/or CO, respectively, represented as:

(2a) $$Zn+H_{2}O\to ZnO+H_{2}$$

(2b) $$Zn+CO_{2}\to ZnO+CO$$

The resulting ZnO is recycled to the first step to close the material cycle, with net reactions H_2_O→H_2_+½O_2_ and CO_2_→CO+½O_2_. The products are produced in separate steps, thereby eliminating their high temperature separation. Since the steps are decoupled, the production of H_2_ and/or CO can be carried out on demand at convenient sites and independent of solar energy availability.

Second-law analyses have been performed to determine theoretical maximum cycle efficiencies and identify sources of irreversibility. Thermogravimetric analyses (TGA) have been performed to identify reaction mechanisms and determine kinetic rate laws for all pertinent reactions. These studies provided the foundation for reactor design, fabrication, testing, modeling, and optimization. Life cycle assessments and economic analyses have been performed to quantify realistic specific CO_2_ emissions and ascertain the competitiveness of the cycle *vis-à-vis* other fuel processing technologies. The bulk of the work is divided up over several years and contained in numerous publications. The aim of this review paper is to consolidate the pertinent findings and technologies and provide an overview of the state-of-the art.

## 2. Second-Law Analyses

**Figure 2:**
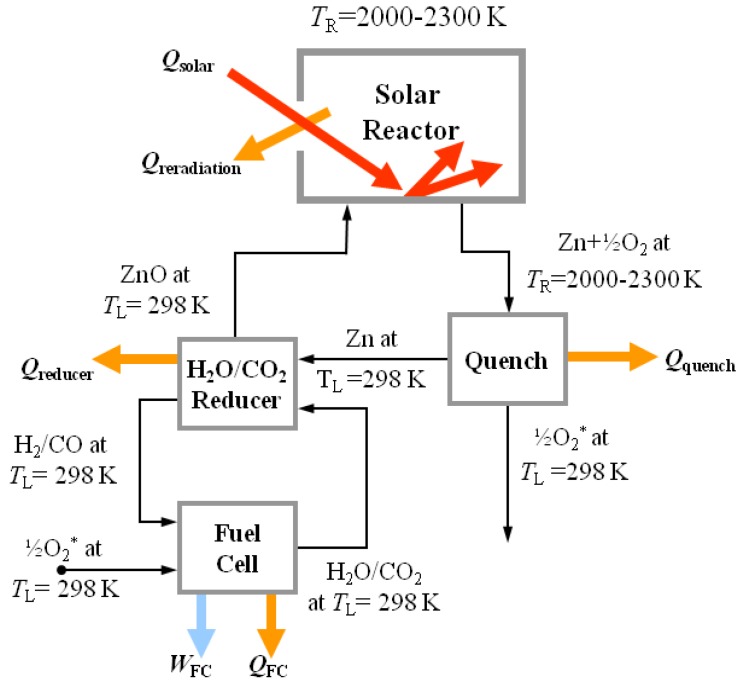
Model flow diagram for second-law analysis of the two-step H_2_O/CO_2_ solar thermochemical cycle based on Zn/ZnO redox reactions.

Second-law (exergy) analyses were performed to establish theoretical maximum solar-to-chemical energy conversion efficiencies. A flow diagram for a generic H_2_O/CO_2_ splitting cycle is shown schematically in Figure 2, comprised of a solar reactor, a quench unit, and a H_2_O/CO_2_ reducer. Heat exchangers for recovering sensible and/or latent heat from the hot products exiting the solar reactor are omitted from consideration. Additional assumptions include: the solar reactor is a perfectly insulated blackbody cavity-receiver (no convection or conduction heat losses; effective absorptivity and emissivity approaching unity); all products separate naturally without expending work; kinetic and potential energies are negligible; and reactions go to completion. The solar reactor is operated in the range 2,000–2,300 K, as the thermal dissociation of ZnO proceeds at reasonable rates above 2,000 K. Lower pressures and/or a carrier gas shift the thermodynamic equilibrium to favor the reaction at lower temperatures.

The solar energy absorption efficiency is defined as the net rate at which solar energy is being absorbed, divided by the solar power input through the solar reactor aperture given by:

(3) $$\eta_{\text{absorption}}=\frac{Q_{\text{reactor, net}}}{Q_{\text{solar}}}=\frac{\dot{n}\Delta H_{\text{ZnO at }T_{\text{L}}\to \text{Zn+}\frac{1}{2}{\text{O}}_{2}\text{ at }T_{R}}}{Q_{\text{solar}}}=1-\left(\frac{\sigma T_{\text{R}}^{{}^{4}}}{IC}\right)$$

*η*_absorption_ expresses the capability of a solar reactor to absorb incoming concentrated solar energy but does not include the losses incurred in collecting and concentrating solar energy. The solar‑to‑chemical energy conversion efficiency is defined as the portion of solar energy input that is converted into chemical energy given by the Gibbs free energy of the products, $-\dot{n}\Delta G$, *i.e.,* the rate of maximum possible work that can be extracted from the products when transformed back to the reactants at *T*_L_ = 298 K in a reversible, ideal fuel cell, *W*_FC,ideal_:

(4) $$\eta_{\text{solar-to-chemical}}=\frac{-\dot{n}\Delta G_{{\text{CO/H}}_{2}+\frac{1}{2}{\text{O}}_{2}\to {\text{CO}}_{2}{\text{/H}}_{2}\text{O at }T_{\text{L}}}}{Q_{\text{solar}}}=\frac{W_{\text{FC,ideal}}}{Q_{\text{solar}}}$$

where $\dot{n}$ represents the molar flow rate. Cycle analysis for splitting H_2_O [15] and CO_2_ [16] at *T*_R_ = 2,000 K (adjusted for comparison with *T*_R_ = 2,300 K [15]), *C* = 5,000 suns, and *I* = 1 kW/m^2^ yields *η*_solar‑to‑chemical_ = 35% and 39%, respectively. Partial recovery of the sensible and latent heat of the products exiting the solar reactor can further boost *η*_solar‑to‑chemical_ to over 50%.

## 3. Kinetic Analyses

The identification of the reaction mechanism for each step and the determination of the pertinent kinetic rate laws are crucial for reactor engineering design.

*ZnO thermal dissociation—*A solar-driven thermogravimeter, in which a packed bed of ZnO particles was directly exposed to concentrated solar radiation at *C* = 2,400 suns, was used to measure the thermal dissociation rate in a set-up closely approximating the heat and mass transfer characteristics of solar reactors [19]. Isothermal runs were performed in the range 1,800–2,100 K and fitted to a zero-order Arrhenius rate law:

(5) $$\frac{1}{A}\frac{dm_{\text{Zn}}}{dt}=k_{0}\exp\left(\frac{-E_{\text{a}}}{RT}\right)$$

where *A* is the packed-bed area exposed to concentration solar irradiation and *T* is the ZnO surface temperature measured by solar-blind pyrometry. The corresponding apparent activation energy, *E*_a_ = 361 ± 53 kJ mol^‑1^, and pre-exponential factor, *k*_0_ = 14.03 × 10^6^ ± 2.73 × 10^6^ kg m^−2^ s^−^^1^, were determined from linear regression with 95% confidence interval. Application of a kinetic expression, derived by L’vov for solid oxides decomposition along with a convective mass transport correlation, yielded kinetic parameters in close agreement with those derived from experimental data [19]. The values for *E*_a_ were comparable to non-solar thermogravimetric [20,21] and solar non-gravimetric [22] analyses. Kinetic parameters determined in an aerosol flow reactor resulted in a pre-exponential factor three orders of magnitude faster than in stationary reactors [21,23].

*Zn oxidation with H*_2_*O—*TGA in the range 623–773 K was performed for Zn oxidation with H_2_O, Equation (2a), with faster reaction rates and higher conversions obtained above the Zn melting point of 693 K [19]. Two distinct reaction mechanisms were identified for submicron-sized Zn particles at 603–633 K with 10–50% H_2_O-Ar [24]: A fast interface-controlled regime followed by a transition to a slow diffusion-controlled regime, limited by Zn ion migration across the ZnO surface layer. A power rate law with an Arrhenius-type temperature dependency was used to describe the surface-controlled regime, given as:

(6) $$\frac{1}{S}\frac{dn_{\text{ZnO}}}{dt}=y_{{\text{H}}_{\text{2}}\text{O}}^{\alpha }k_{0}\exp\left(\frac{-E_{\text{a}}}{RT}\right)$$

where *S* is the total surface area of particles, *n* is the molar amount of ZnO, and *T* represents the temperature measured by a thermocouple in contact with a crucible. Linear regression analysis and a logarithmic transformation yielded *α* = 0.5, *k_0_* = 2 × 10^‑5^ mol cm^−^^2^ s^−1^, and *E_a_* = 42.8 ± 7.4 kJ mol^‑1^. A parabolic rate law with an Arrhenius-type temperature dependency was used to describe the diffusion‑controlled regime, given as:

(7) $$\frac{dm_{\text{ZnO}}}{dt}=\frac{M_{\text{ZnO}}S^{2}}{\rho_{\text{ZnO}}}\frac{k_{0}\exp\left(-\frac{E_{\text{a}}}{RT}\right)}{m_{\text{ZnO}}}$$

resulting in *k*_0_ = 1.5 × 10^‑13^ mol cm^‑1^ s^‑1^ and *E_a_* = 42.9 ± 6.5 kJ mol^‑1^. No dependency upon *y_H_2O__* was observed. Application of a model-free iso-conversional method to non-isothermal TGA with 158 nm Zn particles yielded *E*_a_ = 132 ± 27 kJ mol^‑1 ^ [25]. A similar study using Zn nanoparticles fitted isothermal and non-isothermal hydrolysis experimental data to a kinetic model with *E*_a_ = 87 ± 7 kJ mol^‑1^ and *α* = 3.5 ± 0.5 [26]. Complete Zn-to-ZnO conversions were obtained by TGA for Zn particles smaller than 11 μm [27], with strong dependence on the heating rate and weak dependence on the H_2_O partial pressure.

*Zn oxidation with CO_2_*—Isothermal and non-isothermal TGA runs were performed for Zn oxidation with CO_2_, Equation (2b) [28]. Similar mechanisms to that for Zn reactions with H_2_O were identified: an interface-controlled regime that transitions to a diffusion-controlled regime. The shrinking core model (SCM) and a power rate law with an Arrhenius-type dependency was used to describe the interface-controlled regime, given as:

(8) $$\frac{d}{dt}\left(\frac{n}{S}\right)=y_{{\text{CO}}_{\text{2}}}^{\alpha }k_{0}\exp\left(-\frac{E_{\text{a}}}{RT}\right)$$

with *y_CO_2__* = 0.17–0.75. The kinetic parameters *α* = 0.339 ± 0.034, *k_o_* = 3.61 ± 1.09 mol cm^−2^ s^‑1^, and *E_a_* = 113.6 ± 1.9 kJ mol^‑1^, were determined using non-linear regression analysis and 95% confidence interval for each parameter calculated with constant chi-squared boundaries and covariance matrices. The parabolic rate law used for the diffusion-controlled regime is given as:

(9) $$\frac{d}{dt}\left(\frac{n}{S}\right)=\frac{\rho_{ZnO}^{2}k_{0}\exp\left(-\frac{E_{\text{a}}}{RT}\right)}{2M_{\text{ZnO}}M_{\text{O}}\left(n/S\right)}$$

The kinetic parameters were determined as *k_o_* = 1.04 × 10^‑2^ ± 4.26 × 10^‑2^ cm^2^ s^‑1^ and *E*_a_ = 162.3 ± 25.3 kJ mol^‑1^, respectively, and no dependency upon *y*_CO__2_ was observed.

*Zn oxidation with H_2_O and CO_2_*—The simultaneous reaction between H_2_O and CO_2_ with Zn, Equations (2a) and (2b), was studied for the production of syngas. Preliminary non-isothermal TGA runs at 673–1,423 K and mixtures of 20–90% CO_2_-H_2_O for 7.9 μm Zn particles indicated a strong dependency between the molar ratio of the input gases, H_2_O/CO_2_, and that of the syngas product, H_2_/CO [29]. As a follow-up work, isothermal TGA runs were preformed for reactions with Zn in the temperature range 673–748 K and CO_2_/H_2_O concentrations of 2.5–15% in Ar [30]. The reaction mechanism was characterized by an initial fast interface-controlled regime followed by a slower diffusion-controlled regime. A rate law of Langmuir-Hinshelwood type, encompassing the adsorption of CO_2_/H_2_O on active surface sites, dissociation to atomic O and CO/H_2_, and finally the desorption of CO/H_2_, was formulated to describe the competitiveness of the reactions for the interface-controlled regime. By applying a SCM, the rate law is given as:

(10) $$\frac{d}{dt}\left(\frac{n_{\text{ZnO}}}{S}\right)=\frac{k_{3}(k_{1}y_{{\text{CO}}_{\text{2}}}+k_{2}y_{{\text{H}}_{\text{2}}\text{O}})}{k_{3}+k_{1}y_{{\text{CO}}_{\text{2}}}+k_{2}y_{{\text{H}}_{\text{2}}\text{O}}}$$

The kinetic parameters of *k_i_*, assumed to obey an Arrhenius-type temperature dependency $k=k_{0}\exp\left[E_{\text{a}}/\left(RT\right)\right]$, are listed in Table 1 and were determined with non-linear regression analysis along with 95% confidence intervals.

**Table 1:** Arrhenius kinetic parameters for Equation (10) [30].

rate constant	*k*_0_, mol cm^‑2^ s^‑1^	*E*_a_, kJ mol^‑1^
*k*_1_	0.0018 ± 0.0009	60.3 ± 3.3
*k*_2_	0.29 × 10^−^^5^ ± 0.083 × 10^−^^5^	15.0 ± 1.6
*k*_3_	0.0216 ± 0.0214	70.3 ± 8.4

## 4. Solar Reactor Technology

*ZnO thermal dissociation*—Solar reactors for high-temperature gas-solid thermochemical reactions often feature cavity-receivers containing reacting particles directly exposed to concentrated solar irradiation [31,32]. The direct solar irradiation of the chemical reactants provides efficient energy transfer to the reaction site and bypasses the limitations imposed by indirect heat transfer—by conduction—through reactor walls, *i.e.,* limitations imposed by the materials of the absorber with regard to maximum operating temperature, inertness to the chemical reaction, thermal conductivity, radiative absorptance, and resistance to thermal shocks. The solar energy conversion efficiency is determined by the interrelationship between the solar flux intensity, chemical kinetics of the reaction, and the rate of feeding reactants and recovering products [17]. Additionally, the gaseous products Zn(g) and O_2_ must be cooled rapidly enough to avoid recombination and ensure high Zn yields [33].

Exploratory experimentation to thermally dissociate ZnO was carried out at the solar furnaces and high-flux solar simulators of PSI/ETH (Switzerland) and CNRS-Odeillo/Nancy (France). Initially, small-scale solar reactors containing ZnO pellets or powders within transparent enclosures were directly subjected to high-flux solar radiation while product Zn(g) was condensed on cold surfaces or collected downstream in a filter [34,35,36,37]. Scalable solar reactor concepts have been realized with packed-bed reactors—such as rotary cavity-receivers—and aerosol flow reactors. The “slope” packed-bed reactor consisted of a chamber partly filled with pressed ZnO powder forming a 45° slope that was directly exposed to concentrated solar radiation entering through a transparent quartz window [38]. The uppermost ZnO layers were dissociated, whereas the non-reacted ZnO bed insulated the reactor walls. The reactor was used to identify the kinetic rate law in the range 1,950‑2,400 K [22]. An aerosol flow reactor was tested in the range 1,873–2,023 K using an electrically heated transport tube apparatus [23]. A cooling lance was used to limit Zn(g)/O_2_ recombination, resulting in maximum and mean Zn yields of 18% and 8%, respectively, for residence times of less than 1.8 s.

**Figure 3:**
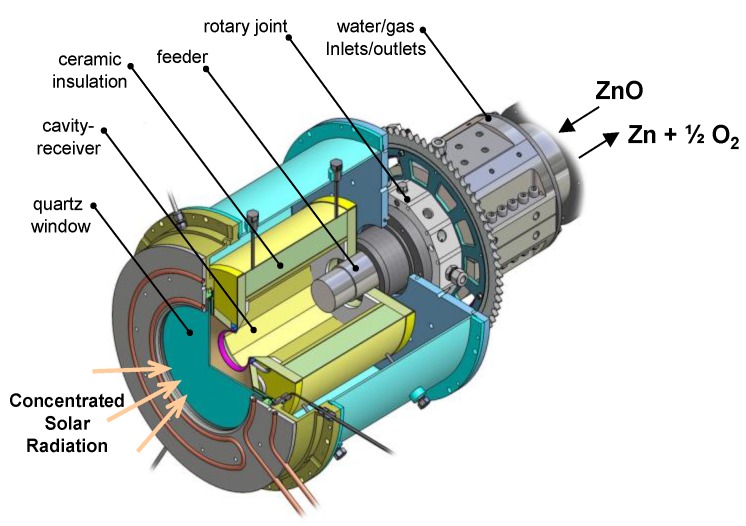
Schematic of the solar rotary reactor configuration [40].

A series of rotary reactors was designed [33,39,40,41]. The solar reactor configuration is shown schematically in Figure 3 [40]. ZnO particles are introduced into a rotating cavity and forced against the cavity walls by centripetal acceleration, creating a packed-bed layer that is directly exposed to concentrated solar irradiation entering the cavity aperture through a transparent quartz window. With this arrangement, the ZnO particles serve simultaneously the functions of radiative absorbers, thermal insulators, and chemical reactants. The reactor has a dynamic feeder that extends and retracts within the cavity, allowing for batches of an evenly distributed layer of ZnO particles of desired thickness over the entire cavity surface. 3D computational fluid dynamics (CFD) was employed to determine the optimal flow configuration for an aerodynamic protection of the quartz window against condensable Zn(g). A 10 kW reactor prototype was fabricated, consisting of a 160 mm-dia. 230 mm-length cylindrical cavity containing a 60 mm-dia. aperture with a 3 mm-thick quartz window. The multi-layer cylindrical cavity was made of sintered ZnO tiles placed on top of a porous 80%Al_2_O_3_–20%SiO_2_ insulation and reinforced by a 95%Al_2_O_3_–5%Y_2_O_3_ ceramic matrix composite, providing mechanical, chemical, and thermal stability and a diffusion barrier for product gases. Experimentation was carried out with the 10 kW reactor subjected to mean C > 3,000 suns and peak C = 5,880 suns [40]. All reactor components performed well at operating temperatures in the range 1,807–1,907 K, measured behind the ZnO tiles. The reactor was operated in a transient ablation mode with semi-continuous feed cycles of ZnO particles, characterized by a rate of heat transfer—predominantly by radiation—to the layer of ZnO particles undergoing endothermic dissociation that proceeded faster than the rate of heat transfer—predominantly by conduction—through the cavity walls.

Reactor modeling has enabled a more comprehensive and in-depth understanding of the competing mechanisms and the identification of major sources of energy losses, which lead to optimization and scaling up. Various aspects of solar chemical reactors have been modeled at different levels of complexity. CFD was used to determine the flow patterns in a tubular aerosol flow reactor for aspect ratios between 0.15–0.45 at 1,900–2,300 K [42]. The overall temperatures were found to decrease with increasing tube diameter, resulting in slower kinetics and lower ZnO-to-Zn conversions. CFD was also used to simulate the thermal reduction of metal oxides in particle-laden flows, including ZnO [43]. In a separate study, a cavity receiver containing a tubular absorber was coupled to a heat transfer model to predict temperatures and ZnO-to-Zn conversions [44]. A more fundamental approach was used to describe the transient radiative transfer of semitransparent ZnO particles undergoing thermal dissociation [45]. The effect of sintering and shrinkage on the rate of heat transfer was examined for a packed bed of ZnO particles subjected to *C* = 1,225–2,133 suns and surface temperatures in the range 1,834–2,109 K [46,47]. Operating conditions were typical of an ablation regime controlled by the rate of radiative heat transfer to the first layers of ZnO undergoing endothermic dissociation. This analysis served to determine the effective thermal transport properties of the ZnO packed bed, namely the extinction coefficient, the surface absorptivity, and the effective thermal conductivity. For simulating the rotary reactor, Figure 2, a heat transfer model was initially formulated [48,49] and then expanded to consider the exact 3D geometry of the cavity, the actual semi-batch feeding mode of ZnO particles, and the fact that the packed-bed layer of ZnO particles lining the cavity shrinks as the reaction progresses [50]. The more accurate kinetic rate law derived from experimental measurements in a solar-driven thermogravimeter for directly irradiated ZnO samples, Equation (5) [19], was incorporated into the model. Radiative transport was coupled to chemical kinetics, which enabled consideration of directional and wavelength depended radiation exchange by Monte Carlo simulations [51,49]. The unsteady-state energy conservation equation that links conductive, convective, and radiative heat transfer to the rate of the reaction is given by:

(11) $$\rho c_{p}\frac{\partial T}{\partial t}=\text{​}\nabla \left(k_{\text{eff}}\nabla T\right)+{q^{\prime \prime \prime }}_{\text{chemistry}}$$

${q^{\prime \prime \prime }}_{\text{chemistry}}$ is the volumetric heat sink rate due to the endothermic dissociation of ZnO,

(12) $${q^{\prime \prime \prime }}_{\text{chemistry}}=-r^{\prime \prime \prime }\Delta H_{\text{r}}\left(T\right)$$

where the enthalpy change of the reaction is determined by [52] and the reaction rate $r^{\prime \prime \prime }$ (in kg m^‑3^s^‑1^) is modeled by applying a zero-order Arrhenius-type rate law, Equation (5). The effective thermal conductivity is given by the sum of the conductive and radiative contributions, using the Rosseland diffusion approximation for the optically-thick packed-bed of ZnO. The radiosity and Monte Carlo methods were applied to obtain the distribution of net radiative fluxes, and the finite volume method with the explicit Euler time integration scheme were applied to solve the integrated form of Equation (11) over a shrinking domain. Experimental validation was accomplished with the 10 kW reactor prototype in terms of temperatures and reaction extents for solar experimental runs with multiple feed-cycles. The ZnO dissociation reaction occurred in the topmost layers which is typical of an ablation regime, as radiative transfer to the endothermic reaction proceeded at a faster rate than heat conduction across the ZnO layer and the insulation. Scaling up the reactor to 100 and 1,000 kW nominal solar power input has the potential of reaching maximum solar-to-chemical conversion efficiencies exceeding 50%, mainly as a result of higher reaction rates at higher operating temperatures and a reduction in the conduction losses through optimization of the geometry to minimize water‑cooled components [50].

*Zn/O_2_ separation technology—*The efficient separation of gaseous products, Zn(g) and O_2_, to avoid recombination upon cooling is an important aspect for the technical realization of the Zn/ZnO cycle. Research has focused on diluting and rapidly cooling of gaseous products below the Zn saturation and solidification points. The condensation of zinc vapor in the presence of O_2_ was studied by fractional crystallization in a temperature-gradient tube furnace [53]. It was found that the oxidation of Zn is a heterogeneous process and, in the absence of nucleation sites, Zn(g) and O_2_ can coexist in a meta-stable state. In particular, the quench efficiency is sensitive to the dilution ratio of Zn(g) in an inert gas flow and to the temperature of the surface on which the products are quenched. Alternatively, electrolytic methods have been proposed for *in situ* separation of Zn(g) and O2 at high temperatures, and experimentally demonstrated to work in small scale reactors [54].

**Figure 4:**
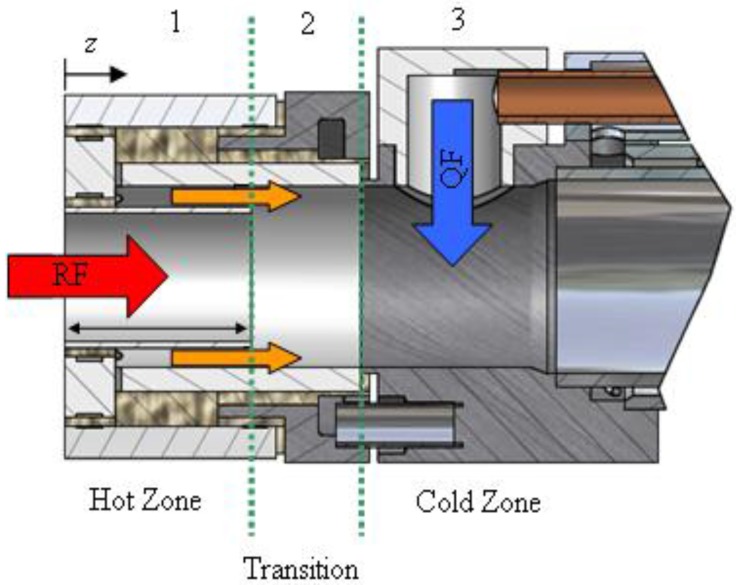
Schematic of the quench apparatus illustrating the three temperature zones [57].

Initial experimentation to rapidly quench thermally dissociated products of ZnO resulted in a global molar efficiency of 60% recovery of the Zn from ZnO [35]. A more mechanistic approach was used to identify rate controlling factors based on temperatures for condensation and solidification of Zn with Ar dilution over a range of partial pressures [37]. A mass transfer model accounting for radial diffusion in the laminar flow regime revealed that the Zn(g) oxidation rate was limited by the gas and wall temperatures below ZnO-decomposition and above Zn(g)-condensation temperatures [55]. A quenching unit was incorporated at the exit of the 10 kW solar reactor to rapidly decrease temperature by injecting Ar into a water-cooled annular outlet [56]. XRD analysis along with SEM and TEM images of the solid products deposited at the quenching zone revealed the formation of spherical particles of sizes in the 0.1–30 μm range, with their surface covered with smaller edged structures, which are characteristic of Zn(g) undergoing condensation followed by Zn(l)/Zn(s) oxidation and coalescence. An optimized quench apparatus attached to a solar-driven thermogravimeter was used to rapidly cool the products resulting from the ZnO decomposition at 1,820–2,050 K [57]. This apparatus, shown schematically in Figure 4, was composed of three distinct zones: 1) A hot zone directly attached to the reactor exit, where the temperature was above the ZnO decomposition temperature, and Zn(g) oxidation was thermodynamically unfavorable; 2) a transition zone where the temperature was below the decomposition temperature of ZnO, but above the saturation temperature of Zn where Zn(g) and O_2_ formed a meta-stable mixture and ZnO can form on surfaces; and 3) a cold zone where the temperature was below the Zn saturation temperature and Zn(g) nucleated homogeneously, forming particles that served as nucleation sites in addition to the surfaces. An annular Ar flow in the second, transition zone was employed for suppressing diffusion of Zn(g) towards the walls. The product gases Zn(g) and O_2_ were quenched by injection of Ar in the third, cold zone at cooling rates from 20,000 to 120,000 K s^‑1^, suppressing the formation of ZnO in the gas phase and at the walls. Zinc content of the collected particles downstream varied in the range 40–94% for Ar/Zn(g) dilutions of 170 to 1,500 [57]. Quenching units were also modeled to determine ideal operating conditions [58]. An aerosol kinetic model predicted an increase in the Zn yield upon dilution prior to fast quenching to initiate nucleation [59].

*H_2_/CO generation***—**Aerosol flow reactors were designed for the formation of Zn nanoparticles followed by their *in situ* reaction with H_2_O and CO_2_. The advantages of using Zn nanoparticles are three-fold: (1) Their inherent high specific surface area augments the reaction kinetics, heat transfer, and mass transfer; (2) their large surface to volume ratio favors complete or nearly complete oxidation; and (3) their entrainment in a gas flow allows for continuous feeding of reactants and removal of products. Tubular aerosol flow reactors featured three zones for Zn evaporation, steam quenching, and Zn/H_2_O reaction, respectively [60,61,62,63,64]. Superheated Zn vapor carried by Ar was mixed and quenched by a superheated, equimolar H_2_O/Ar stream resulting in Zn/ZnO nanoparticles and H_2_. When the Zn evaporation zone was operated at 1,023 K and the Zn/H_2_O reaction zone was operated continuously just below the Zn(g) saturation temperature, Zn particles of 69 nm average crystallite size were formed and *in situ* hydrolyzed by up to 83% degree of chemical conversion, while the H_2_ yield reached up to 70% after a single pass of H_2_O of 0.85 s residence time [60,61]. Follow-up studies with an improved quenching yielded up to 90% H_2_ conversion and nanoparticles with Zn and ZnO mean crystallite sizes of 100 and 40 nm, respectively, containing up to 80 wt% ZnO [62]. Quench rates of 2–6 × 10^4^ K s^‑1^ yielded conversions of up to 95% at the expense of low particle yield due to significant wall deposition with subsequent hydrolysis [63]. Aerosol particles with hexagonal structure were formed by Zn evaporation-condensation containing low ZnO mass fraction. In contrast, operation at quench rates up to 10^6^ K s^−^^1^ led to increased particle yield but lower conversion. Filamentary and rod-like particles were formed with high ZnO content by surface reaction and coagulation [63]. Conversions in the range 87–96% were obtained in a similar setup at 1,023–1,073 K with residence times varying from 1.7–2.1 s [64]. Zn particles with an average size of 158 nm were reacted with steam in an aerosol flow tube reactor to yield 24% conversion at 813 K with a residence time of 0.6 s [63]. High Zn-to-ZnO conversions were also obtained for reducing CO_2_ to CO over short residence times, e.g., up to 88% over 3 s [65]. These aerosol flow configurations offered high Zn-to-ZnO conversions over short residence times due to augmented reaction kinetics and heat/mass transfer. However, the reactions primarily occurred heterogeneously outside the aerosol jet flow on surfaces with Zn deposition, making the ZnO recovery, process control and scale-up problematic.

Batch reactors have been applied to reduce both CO_2_ and H_2_O with Zn. A batch reactor was used to examine the influence of pre-heated steam in the range 473–823 K reacting with Zn particles obtained from the solar carbothermic reduction of ZnO [66], yielding Zn-to-ZnO conversions in the range 24–81% [67]. Laboratory studies were performed with steam bubbling through molten Zn in the range 723–773 K [68], but the continuous removal of ZnO(s) was thought to pose process complications. An alternative approach considered the reaction of Zn vapor with H_2_-H_2_O vapor mixtures in a continuous flow at 773–1,173 K [69], but oxidation occurred heterogeneously on the silica reactor walls by vapor deposition, making efficient recovery of ZnO difficult. The reaction kinetics was further inhibited by the creation of a ZnO layer around un-reacted Zn particles. Recently, a novel packed-bed reactor of Zn/ZnO particles for CO_2_ reduction was demonstrated with Zn-to-ZnO conversions as high as 71% [70]. The mixture of Zn and ZnO particles offered an effective inert support for preventing sintering and enabled simple and complete recovery of ZnO particles for recycling to the first solar step.

## 5. Life Cycle and Economic Analyses

A well-to-wheel life cycle assessment was conducted to quantify greenhouse gas emissions and cumulative energy demand for solar hydrogen production, transport, and usage in future passenger car transportation [71]. Solar hydrogen production methods were compared with other production technologies selected, namely: nuclear and hydropower-based electrolysis, steam methane reforming, and coal gasification. Utilization of hydrogen in fuel cells was compared with advanced gasoline and diesel powertrains. Solar scenarios show distinctly lower greenhouse gas (GHG) emissions than fossil‑based scenarios. For example, using solar hydrogen in fuel cell cars reduces life cycle GHG emissions by 70% compared to advanced fossil fuel powertrains and by more than 90% if car and road infrastructure are not considered. Solar hydrogen production allows a reduction of fossil energy requirements by a factor of up to 10 compared to using conventional technologies. Major environmental impacts are associated with the construction of the steel-intensive infrastructure for solar energy collection due to mineral and fossil resource consumption as well as discharge of pollutants related to today’s steel production technology. Despite high infrastructure demands and associated environmental impacts, solar energy stored in H_2_ and used in automobiles was found to be a promising option for substituting fossil-based fuels in sustainable future transportation systems. Economic analyses have been carried out to determine the long-term potential of the Zn/ZnO-cycle realized in a solar tower system. The cost of H_2_ ranged between 0.10 and 0.15 $/kWh (based on its LHV and a heliostat field cost at 100 to 150 $/m^2^), and thus might become competitive *vis-à-vis* other paths for producing solar H_2_ from H_2_O [15,72,73,74]. Credit for pollution abatement and CO_2_ mitigation can accelerate the deployment of the solar thermochemical technology. A comparison of H_2_ produced via steam methane reforming and the Zn/ZnO cycle concluded that a significantly higher carbon tax is required to make the Zn/ZnO competitive than is likely to be implemented [75]. Therefore, the economic viability of the Zn/ZnO cycle must also include competitive, incentive policies that lead to early implementation of solar H_2_ plants. On the other hand, the Zn/ZnO cycle can be applied to split both H_2_O and CO_2_ and produce both H_2_ and CO, thereby laying the path to the solar production of synthetic liquid hydrocarbons for fueling the transportation sector and the existing massive global infrastructure.

## 6. Summary and Conclusions

We have outlined the systematic approach to realize the science and technology required to produce H_2_ and/or CO in two-step H_2_O/CO_2_ splitting thermochemical cycles via Zn/ZnO redox reactions. Second-law analyses indicate the potential of achieving high solar-to-chemical energy conversion efficiencies and, consequently, economic competitiveness *vis-à-vis* other routes for producing solar fuels from H_2_O and CO_2_. Thermogravimetry was used to identify reaction mechanisms and determine kinetic parameters for all reactions considered. The feasibility of the solar reactor technology has been experimentally demonstrated with a 10 kW solar reactor prototype. Further research and development towards optimization and scale-up is warranted.

## References

[1] Fletcher E.A., Moen R.L. (1977). Hydrogen and oxygen from water. Science.

[2] Bilgen E., Ducarroir M., Foex M., Sibieude F., Trombe F. (1977). Use of solar energy for direct and two-step water decomposition cycles. Int. J. Hydrogen Energ..

[3] Traynor A.J., Jensen R.J. (2002). Direct solar reduction of CO_2_ to fuel: First prototype results. Ind. Eng. Chem. Res..

[4] Price R.J., Morse D.A., Hardy S.L., Fletcher T.H. (2004). Modeling the direct solar conversion of CO_2_ to CO and O_2_. Ind. Eng. Chem. Res..

[5] Price R.J., Fletcher T.H., Jensen R.J. (2007). Using computational fluid dynamics modeling to improve the performance of a solar CO_2_ converter. Ind. Eng. Chem. Res..

[6] Steinfeld A. (2005). Solar thermochemical production of hydrogen—A review. Sol. Energy.

[7] Perkins C., Weimer A.W. (2009). Solar-thermal production of renewable hydrogen. AIChE J..

[8] Abanades S., Charvin P., Flamant G., Neveu P. (2006). Screening of water-splitting thermochemical cycles potentially attractive for hydrogen production by concentrated solar energy. Energy.

[9] Kodama T. (2003). High-temperature solar chemistry for converting solar heat to chemical fuels. Prog. Energ. Combust..

[10] Kodama T., Gokon N. (2007). Thermochemical cycles for high-temperature solar hydrogen production. Coordin. Chem. Rev..

[11] Fletcher E.A. (2001). Solarthermal processing: A review. J. Sol. Energy Eng..

[12] Inoue M., Hasegawa N., Uehara R., Gokon N., Kaneko H., Tamaura Y. (2004). Solar hydrogen generation with H_2_O/ZnO/MnFe_2_O_4_ system. Sol. Energy.

[13] Miller J.E., Allendorf M.D., Diver R.B., Evans L.R., Siegel N.P., Stuecker J.N. (2008). Metal oxide composites and structures for ultra-high temperature solar thermochemical cycles. J. Mater. Sci..

[14] Chueh W.C., Haile S.M. (2009). Ceria as a thermochemical reaction medium for selectively generating syngas or methane from H_2_O and CO_2_. ChemSusChem.

[15] Steinfeld A. (2002). Solar hydrogen production via a two-step water-splitting thermochemical cycle based on Zn/ZnO redox reactions. Int. J. Hydrogen Energ..

[16] Gálvez E., Loutzenhiser P., Hischier I., Steinfeld A. (2008). CO_2_ splitting via two-step solar thermochemical cycles with Zn/ZnO and FeO/Fe_3_O_4_ redox reactions: Thermodynamic analysis. Energ. Fuel..

[17] Palumbo R., Lédé J., Boutin O., Elorza-Ricart E., Steinfeld A., Möller S., Weidenkaff A., Fletcher E.A., Bielicki J. (1998). The production of Zn from ZnO in a single step high temperature solar decomposition process I. The scientific framework for the process. Chem. Eng. Sci..

[18] Perkins C., Weimer A.W. (2004). Likely near-term solar-thermal water splitting technologies. Int. J. Hydrogen Energ..

[19] Schunk L.O., Steinfeld A. (2009). Kinetics of the thermal dissociation of ZnO exposed to concentrated solar irradiation using a solar-driven thermogravimeter in the 1800–2100 K range. AIChE J..

[20] Weidenkaff A., Reller A.W., Wokaun A., Steinfeld A. (2000). Thermogravimetric analysis of the ZnO/Zn water splitting cycle. Thermochim. Acta.

[21] Perkins C., Lichty P., Weimer A.W. (2007). Determination of aerosol kinetics of thermal ZnO dissociation by thermogravimetry. Chem. Eng. Sci..

[22] Möller S., Palumbo R. (2001). Solar thermal decomposition kinetics of ZnO in the temperature range 1950–2400 K. Chem. Eng. Sci..

[23] Perkins C., Lichty P.R., Weimer A. (2008). Thermal ZnO dissociation in a rapid aerosol reactor as part of a solar hydrogen production cycle. Int. J. Hydrogen Energ..

[24] Ernst F.O., Steinfeld A., Pratsinis S.E. (2009). Hydrolysis rate of submicron Zn particles for solar H_2_ synthesis. Int. J. Hydrogen Energ..

[25] Funke H.H., Diaz H., Liang X., Carney C.S., Weimer A.W., Lib P. (2008). Hydrogen generation by hydrolysis of zinc powder aerosol. Int. J. Hydrogen Energ..

[26] Chambon M., Abanades S., Flamant G. (2009). Kinetic investigation of hydrogen generation from hydrolysis of SnO and Zn solar nanopowders. Int. J. Hydrogen Energ..

[27] Lv M., Zhou J., Yang W., Cen K. (2010). Thermogravimetric analysis of the hydrolysis of zinc particles. Int. J. Hydrogen Energ..

[28] Loutzenhiser P.G., Gálvez M.E., Hischier I., Stamatiou A., Frei A., Steinfeld A. (2009). CO_2_ splitting via two-step solar thermochemical cycles with Zn/ZnO and FeO/Fe_3_O_4_ redox reactions II: Kinetic analysis. Energ. Fuel..

[29] Stamatiou A., Loutzenhiser P.G., Steinfeld A. (2010). Solar syngas production via H_2_O/CO_2_-splitting thermochemical cycles with Zn/ZnO and FeO/Fe_3_O_4_ redox reactions. Chem. Mater..

[30] Stamatiou A., Loutzenhiser P.G., Steinfeld A. (2010). Solar syngas production from H_2_O and CO_2_ via two-step thermochemical cycles based on Zn/ZnO and FeO/Fe_3_O_4_ redox reactions: Kinetic analysis. Energ. Fuel..

[31] Palumbo R., Keunecke M., Möller S., Steinfeld A. (2004). Reflections on the design of solar thermal chemical reactors: Thoughts in transformation. Energy.

[32] Steinfeld A., Palumbo R., Meyers R.A. (2001). Solar thermochemical process technology. Encyclopedia of Physical Science and Technology.

[33] Haueter P., Moeller S., Palumbo R., Steinfeld A. (1999). The production of zinc by thermal dissociation of zinc oxide—Solar chemical reactor design. Sol. Energy.

[34] Weidenkaff A., Reller A., Sibieude F., Wokaun A., Steinfeld A. (2000). Experimental investigations on the crystallization of zinc by direct irradiation of zinc oxide in a solar furnace. Chem. Mater..

[35] Elorza-Ricart E., Martin P.Y., Ferrer M., Lédé J. (1999). Direct thermal splitting of ZnO followed by a quench. Experimental measurements of mass balances. J. Phys. IV France.

[36] Elorza-Ricart E., Ferrer M., Lédé J. (2000). Dissociation thermique directe de l’oxide de zinc par voie solaire. Entropie.

[37] Lédé J., Boutin O., Elorza-Ricart E., Ferrer M. (2001). Solar thermal splitting of zinc oxide. A review of some of the rate controlling factors. J. Sol. Energy Eng..

[38] Moeller S., Palumbo R. (2001). The development of a solar chemical reactor for the direct thermal dissociation of zinc oxide. J. Sol. Energy Eng..

[39] Müller R., Haeberling P., Palumbo R.D. (2006). Further advances toward the development of a direct heating solar thermal chemical reactor for the thermal dissociation of ZnO(s). Sol. Energy.

[40] Schunk L., Haeberling P., Wepf S., Wuillemin D., Meier A., Steinfeld A. (2008). A solar receiver-reactor for the thermal dissociation of zinc oxide. J. Sol. Energy Eng..

[41] Chambon M., Abanades S., Flamant G. (2010). Design of a lab-scale rotary cavity-type solar reactor for continuous thermal dissociation of volatile oxides under reduced pressure. J. Sol. Energy Eng..

[42] Perkins C., Weimer A. (2008). Computational fluid dynamics simulation of a tubular aerosol reactor for solar thermal ZnO decomposition. J. Sol. Energy Eng..

[43] Abanades S., Charvin P., Flamant G. (2007). Design and simulation of a solar chemical reactor for the thermal reduction of metal oxides - Case study of zinc oxide dissociation. Chem. Eng. Sci..

[44] Melchior T., Perkins C., Weimer A.W., Steinfeld A. (2008). A cavity-receiver containing a tubular absorber for high-temperature thermochemical processing using concentrated solar energy. Int. J. Therm. Sci..

[45] Dombrovsky L.A., Lipiński W., Steinfeld A. (2007). A diffusion-based approximate model for radiation heat transfer in a solar thermochemical reactor. J. Quant. Spectrosc. Ra..

[46] Schunk L.O., Lipiński W., Steinfeld A. (2009). Ablative heat transfer in a shrinking packed-bed of ZnO undergoing solar thermal dissociation. AIChE J..

[47] Dombrovsky L.A., Schunk L., Lipiński W., Steinfeld A. (2009). An ablation model for the thermal decomposition of porous zinc oxide layer heated by concentrated solar radiation. Int. J. Heat Mass Tran..

[48] Müller R., Steinfeld A. (2007). Band-approximated radiative heat transfer analysis of a solar chemical reactor for the thermal dissociation of zinc oxide. Sol. Energy.

[49] Müller R., Lipiński W., Steinfeld A. (2008). Transient heat transfer in a directly-irradiated solar chemical reactor for the thermal dissociation of ZnO. Appl. Therm. Eng..

[50] Schunk L.O., Lipiński W., Steinfeld A. (2009). Heat transfer model of a solar receiver-reactor for the thermal dissociation of ZnO–validation at 10 kW and scale-up to 1 MW. Chem. Eng. J..

[51] Lipiński W., Thommen D., Steinfeld A. (2006). Unsteady radiative heat transfer within a suspension of ZnO particles undergoing thermal dissociation. Chem. Eng. Sci..

[52] Roine A. (2002). Outokumpu HCS Chemistry 5.11. Outokumpu Research Oy, Pori, Finland.

[53] Weidenkaff A., Steinfeld A., Wokaun A., Eichler B., Reller A. (1999). The direct solar thermal dissociation of ZnO: Condensation and crystallization of Zn in the presence of oxygen. Sol. Energy.

[54] Fletcher E.A. (1999). Solarthermal and solar quasi-electrolytic processing and separations: Zinc from Zinc Oxide as an example. Ind. Eng. Chem. Res..

[55] Keunecke M., Meier A., Palumbo R. (2004). Solar thermal decomposition of zinc oxide—An initial investigation of the recombination reaction in the temperature range 1100–1250 K. Chem. Eng. Sci..

[56] Müller R., Steinfeld A. (2008). H_2_O-splitting thermochemical cycle based on ZnO-Zn-redox—Quenching the effluents from the ZnO dissociation. Chem. Eng. Sci..

[57] Gstoehl D., Brambilla A., Schunk L.O., Steinfeld A. (2008). A quenching apparatus for the gaseous products of the solar thermal dissociation of ZnO. J. Mater. Sci..

[58] Chambon M., Abanades S., Flamant G. (2010). Solar thermal reduction of ZnO and SnO_2_—Characterization of the recombination reaction with O_2_. Chem. Eng. Sci..

[59] Alxneit I. (2008). Assessing the feasibility of separating a stoichiometric mixture of zinc vapor and oxygen by a fast quench—Model calculations. Sol. Energy.

[60] Weiss R.J., Ly H.C., Wegner K., Pratsinis S.E., Steinfeld A. (2005). H_2_ production by Zn hydrolysis in a hot-wall aerosol reactor. AIChE J..

[61] Wegner K., Ly H.C., Weiss R.J., Pratsinis S.E., Steinfeld A. (2006). *In situ* formation and hydrolysis of Zn nanoparticles for H_2_ production by the two-step ZnO/Zn water-splitting thermochemical cycle. Int. J. Hydrogen Energ..

[62] Ernst F.O., Tricoli A., Pratsinis S.E., Steinfeld A. (2006). Co-synthesis of H_2_ and ZnO by in-situ Zn aerosol formation and hydrolysis. AIChE J..

[63] Melchior T., Piatkowski N., Steinfeld A. (2009). H_2_ production by steam-quenching of Zn vapor in a hot-wall aerosol flow reactor. Chem. Eng. Sci..

[64] Abu Hamed T., Venstrom L., Alshare A., Brülhart M., Davidson J.H. (2009). Study of a quench device for simultaneous synthesis and hydrolysis of Zn nanoparticles: Modeling and experiments. J. Sol. Energy Eng..

[65] Loutzenhiser P.G., Gálvez M.E., Hischier I., Graf A., Steinfeld A. (2010). CO_2_ splitting in an aerosol flow reactor via the two-step Zn/ZnO solar thermochemical cycle. Chem. Eng. Sci..

[66] Epstein M., Olalde G., Santén S., Steinfeld A., Wieckert C. (2008). Towards the industrial solar carbothermal production of zinc. J. Sol. Energy Eng..

[67] Vishnevetsky I., Epstein M. (2007). Production of hydrogen from solar zinc in steam atmosphere. Int. J. Hydrogen Energ..

[68] Berman A., Epstein M. (2000). The kinetics of hydrogen production in the oxidation of liquid zinc with water vapor. Int. J. Hydrogen Energ..

[69] Clarke J.A., Fray D.J. (1979). Oxidation of zinc vapour by hydrogen-water vapour mixtures. T. I. Min. Metall. C.

[70] Loutzenhiser P.G., Barthel F., Stamatiou A., Steinfeld A. (2010). CO_2_ reduction with Zn particles in a packed-bed reactor. AIChE J..

[71] Felder R., Meier A. (2008). Well-to-wheel analysis of solar hydrogen production and utilization for passenger car transportation. J. Sol. Energy Eng..

[72] Charvin P., Abanades S., Lemort F., Flamant G. (2008). Analysis of solar chemical processes for hydrogen production from water splitting thermochemical cycles. Energ. Con. Manage..

[73] Graf D., Monnerie N., Roeb M., Schmitz M., Sattler C. (2008). Economic comparison of solar hydrogen generation by means of thermochemical cycles and electrolysis. Int. J. Hydrogen Energ..

[74] Weimer A.W., Perkins C., Lichty P., Funke H., Zartmann J., Hirsch D., Bingham C., Lewandowski A., Haussener S., Steinfeld A. (2009). Development of a Solar-thermal ZnO/Zn Water-splitting Thermochemical Cycle.

[75] Haltiwanger J.F., Davidson J.H., Wilson E.J. (2010). Renewable hydrogen from the Zn/ZnO solar thermochemical cycle: A cost and policy analysis. J. Sol. Energy Eng..

